# Neo Sex Chromosomes, Colour Polymorphism and Male-Killing in the African Queen Butterfly, *Danaus chrysippus* (L.)

**DOI:** 10.3390/insects10090291

**Published:** 2019-09-09

**Authors:** David A.S. Smith, Walther Traut, Simon H. Martin, Piera Ireri, Kennedy S. Omufwoko, Richard ffrench-Constant, Ian J. Gordon

**Affiliations:** 1 Natural History Museum, Eton College, Windsor SL4 6DW, UK; 2 Institut für Biologie, Zentrum für Medionische Struktur-und Zellbiologie, Universität zu Lübeck, Ratzeburger Allee 160, 23538 Lübeck, Germany; 3 Institute of Evolutionary Biology, University of Edinburgh, Edinburgh EH9 3FL, UK; 4 Department of Zoological Sciences, Kenyatta University, Nairobi P.O. Box 43844-00100, Kenya; 5 Department of Ecology and Evolutionary Biology, Princeton University, Princeton, NJ 08544, USA; 6 Mpala Research Centre, Nanyuki P.O. Box 555-10400, Kenya; 7 Centre for Ecology and Conservation, University of Exeter, Penryn Campus, Penryn TR10 9FE, UK; 8 BirdLife International Kigali Office, Kigali Post Office, Kigali P.O. Box 2527, Rwanda

**Keywords:** colour polymorphism, *Danaus chrysippus*, defence, ‘magic trait’, male-killing, mimicry, neo sex chromosomes, resource competition, speciation

## Abstract

*Danaus chrysippus* (L.), one of the world’s commonest butterflies, has an extensive range throughout the Old-World tropics. In Africa it is divided into four geographical subspecies which overlap and hybridise freely in the East African Rift: Here alone a male-killing (MK) endosymbiont, *Spiroplasma ixodetis*, has invaded, causing female-biased populations to predominate. In ssp. *chrysippus*, inside the Rift only, an autosome carrying a colour locus has fused with the W chromosome to create a neo-W chromosome. A total of 40–100% of Rift females are neo-W and carry *Spiroplasma*, thus transmitting a linked, matrilineal neo-W, MK complex. As neo-W females have no sons, half the mother’s genes are lost in each generation. Paradoxically, although neo-W females have no close male relatives and are thereby forced to outbreed, MK restricts gene flow between subspecies and may thus promote speciation. The neo-W chromosome originated in the Nairobi region around 2.2 k years ago and subsequently spread throughout the Rift contact zone in some 26 k generations, possibly assisted by not having any competing brothers. Our work on the neo-W chromosome, the spread of *Spiroplasma* and possible speciation is ongoing.

## 1. Introduction

**Figure 1:**
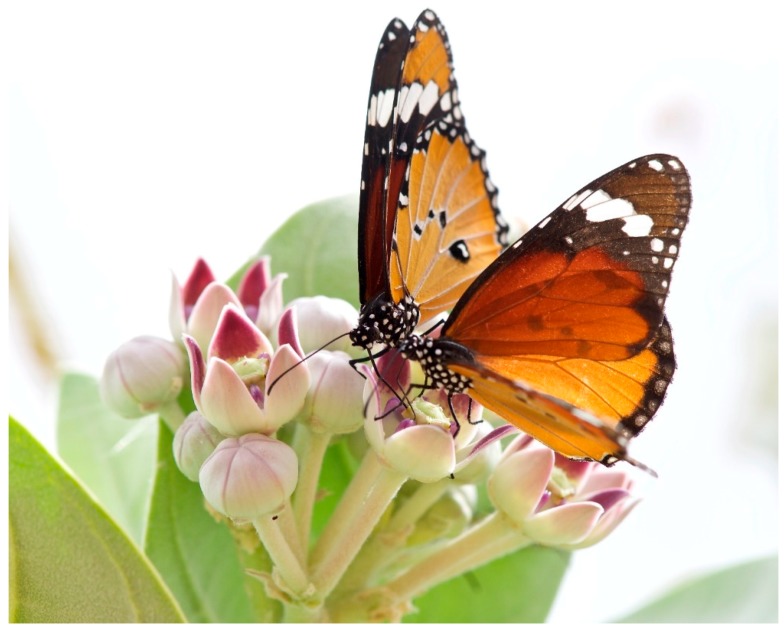
Male (left) and female Queens sharing nectar from a *Calotropis procera* (Aiton) W.T.Aiton flower on Fuerteventura. Photo, Chris Ward.

*Danaus (Anosia) chrysippus* (L.) (Figure 1), is commonly called the African Queen butterfly or African Monarch in Africa and in Asia the Golden Monarch or Plain Tiger. *D chrysippus* is sister to the American Queen, *D. gilippus* (Cramer) [1], rather than the Monarch, *D. plexippus* (L.). Poulton [2] claimed that *D. chrysippus* was the commonest butterfly in the world. Its ubiquity results from, firstly, its extensive geographical spread across the entire Old-World tropics and sub-tropics, extending in summer to warm temperate regions, secondly, the high population densities it attains in favourable locations and, third, in the tropics its short generation time of one month, which combined with maximum longevity in excess of two months, implies that two or more overlapping generations fly together. *D. chrysippus* does not enter closed forest but occurs in most other low level, open habitats ranging from wooded savannah to semi-desert. In historic times *Homo sapiens* has undoubtedly greatly increased available habitat for the butterfly through deforestation, desertification and the proliferation of weedy farms and flower-rich gardens. Furthermore, the African Queen was the first butterfly literally to attain icon status some 3.5 k years ago when it was depicted in a Bronze Age fresco on the tomb of Nebamun, Valley of the Kings, Egypt, and thus became the first butterfly in recorded history [3]. Ever since being first named by Linnaeus in 1758 *D. chrysippus* has retained a high research profile.

Of the 11 *Danaus* species listed by Ackery and Vane-Wright [4], *D. chrysippus* was one of only two recognised by Linnaeus (1758), the other being *D. plexippus*: He assigned both to the omnibus genus *Papilio* in the group ‘Danai festivi’. Over the next 150 years (1759–1909), specific status was claimed for a further nine taxa, eight of which remain generally agreed subspecies of *D. chrysippus* while one (*petilia*) now has specific status [5,6]. Six other varieties now known to be hybrid forms found mainly in Africa, have been described and named (Table 1).

**Figure 2:**
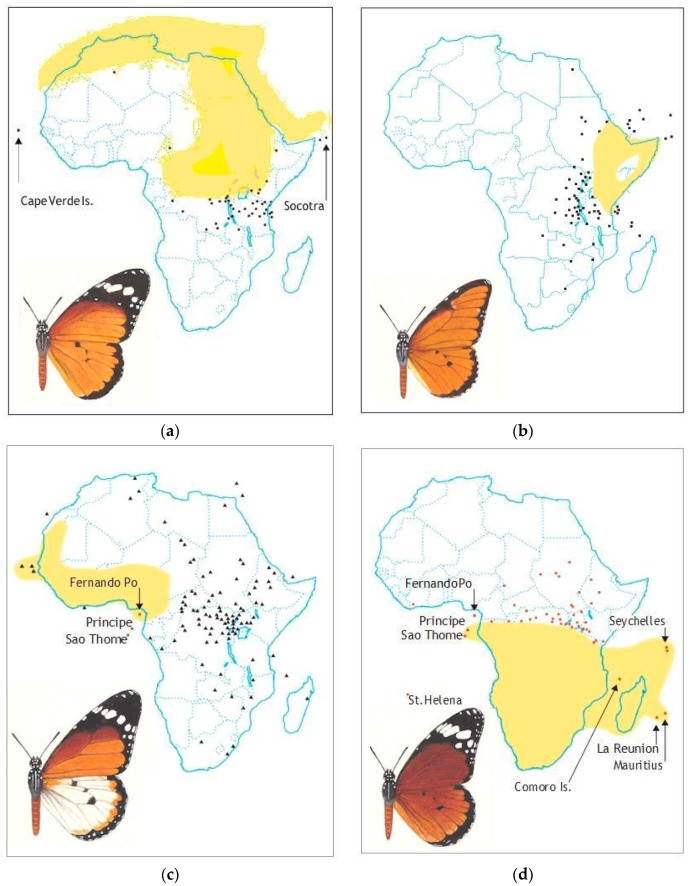
Geographical distribution of the subspecies (ssp.) of *Danaus chrysippus* in Africa. (**a**) ssp. *chrysippus*; (**b**) ssp. *dorippus*; (**c**) ssp. *alcippus*; (**d**) ssp. *orientis*. Shaded areas show the ‘heartlands’ where there is year-round occupancy, whereas the areas outside the shaded area indicate seasonal migration or occasional occurrence. The database comprises some 7000 field records and 15,000 museum specimens [14].

The five Asian subspecies (*chrysippus*, *alcippoides*, *bataviana*, *gelderi* and *cratippus*) replace one another geographically and hybridise only in narrow areas where their ranges abut [8]. Their genetics have apparently not been studied. In contrast, the four African subspecies (*chrysippus*, *dorippus*, *alcippus* and *orientis*) have been subjects of extensive genetic, cytological, molecular, ecological and behavioural studies. Moreover, although allopatric over large swathes of Africa (Figure 2), the subspecies overlap in a substantial area of East and Central Africa [9] which we call the contact zone. Within this zone, all four colour forms coexist and interbreed freely [10,11]. It is for this reason that the contact zone was once thought to be a region where *D. chrysippus* was polymorphic in the conventional sense (genetic polymorphism is the occurrence together in the same locality of two or more discontinuous forms of a species in such proportions that the rarest of them cannot be maintained by recurrent mutation [12]). [10,12]. More recently, several substantial field studies and breeding programmes in East Africa have rejected this interpretation [13,14]. The second focus has been the mimetic association of *D. chrysippus* with *Hypolimnas misippus* (L.), a female-limited polymorphic mimic which breaks most of the rules normally associated with both forms of mimicry, Batesian [15] and Müllerian [16].

## 2. The Record

### 2.1. Host Plants

The larval host plants almost invariably belong to the family Apocynaceae, subfamily Asclepiadoideae (milkweeds), but occasional records include other Apocynaceae subfamilies, Apocynoideae, Secamonoideae and Periplocoideae, and uniquely the family Convolvulaceae [17]. There are some 55 known milkweed hosts [9], and doubtless many more await discovery; they are hugely varied in vegetative form, ranging from cactiform succulents through leafless and leafy climbers to leafy perennial herbs and substantial shrubs. The wide range of larval host plants used by the Queen no doubt underlies its extensive geographical and ecological footprint, and its notorious ability to inhabit new territory; in this respect it resembles a similarly dispersive butterfly, *Vanessa cardui* (L.). Almost every warm island in the Atlantic, Indian and western Pacific Oceans with native or introduced milkweed has been colonised by *D. chrysippus* [9].

### 2.2. Cardenolide Glycosides

Many milkweeds, including the most favoured hosts, contain a bitter-tasting milky sap, rich in C_23_ steroid derivatives, known as cardenolide glycosides (CGs); the sap is not only highly distasteful and emetic but also, being cardioactive, toxic to most potential defoliators. In vivo biosynthesis of CGs is unknown in insects, hence their occurrence in the tissues of all life history stages of *D. chrysippus* and other milkweed insects is invariably plant-derived, either through larvae eating leaves or adults imbibing nectar (Figure 1). Paradoxically, adult *D. chrysippus*, unlike its cousin *D. plexippus* but in line with its sister *D. gilippus*, is often CG-negative even when reared on CG-rich food-plant [18]. However, it is likely that an actively feeding larva with a gut full of CG-rich milkweed or an adult that has imbibed CG-rich nectar (Figure 1) will be defended by its bitter taste against all but experienced predators, i.e., those specialists able to strip off the exoskeleton or skillfully remove the gut of their prey before swallowing. Nonetheless, naïve predators that swallow CG-defended prey experience emesis, an uncomfortable experience they quickly learn to avoid [19,20].

The mystery of the Monarch’s ability to store relatively high concentrations of CGs in its body compared to other congeners has recently been solved [9]. In common with at least six insect species from four orders, Hemiptera, Coleoptera, Diptera and Lepidoptera [21], the α subunit of the trans-membrane enzyme Na^+^/K^+^-ATPase of the Monarch carries two point-mutations which render it insensitive to binding by CGs and thus protects normal Na^+^/K^+^ exchange across cell membranes [22]. Two subspecies of *D. chrysippus*, *orientis* from southern Africa and *chrysippus* from Turkey [18], as well as *D. petilia* and *D. gilippus*, carry one of these mutations, which is apparently shared with all other Danainae, but the other is private to *D. plexippus*, hence the latter’s superior resistance to CGs. Nonetheless, albeit the mean CG content of *D. plexippus* is 1.76‰ (*n* = 4133) and averages some eight times higher than *D. chrysippus*—0.23‰ (*n* = 678)—the latter is notably variable (0.00–1.11‰). As many of the *D. chrysippus* analyses are over 40 years old, the CG content of its several subspecies should be re-visited. Intriguingly, f. alcippus from Dar es Salaam averaged only 0.09‰ compared to 0.33‰ (*n* = 120) for f. dorippus raised as brood mates on *Calotropis gigantea* (L.) Dryand [23]; this difference between sibs suggests that CG storage may be to some extent under genetic control and, therefore, could vary among *D. chrysippus* subspecies. *D. chrysippus* shows a preference for CG-rich host plants. Although CGs stored in the butterfly’s body have been shown to confer protection from avian predators, larval behaviours such as trenching by first instars and petiole-biting by older larvae, which cut off the phloem source of CG-rich milky sap, are universal [24]. Furthermore, it is clear that sticky milky sap sometimes entraps and kills young larvae. The paradox deepens when one considers that CGs have antibiotic properties and yet, in *D. plexippus*, combating their toxicity imposes metabolic costs reflected in smaller body size [25,26]. Thus, it seems the use of milkweed hosts comprises a series of trade-offs, the major advantage, besides protection from avian predators, possibly being that, because large herbivores abhor milkweeds, defoliating larvae are protected both from direct competition from other defoliator species and accidental death from browsing mammals such as goats, donkeys and camels.

### 2.3. Pyrrolizidine Alkaloids

Extrapolating from Monarch studies, until around 1975 CGs were assumed to be the mainline chemical defence of *Danaus* and related genera in the tribe Danaini. However, Rothschild and colleagues [27] opined that most danaines from the subtribes Danaina (*Danaus*, *Tirumala*) and its sister Amaurina (*Amauris*, *Ideopsis*, *Parantica*) preferred or used only milkweed food-plants that lacked CGs [4]. Pliske [28] was the first of many investigators cited in [9] to observe the addiction of male, but not female, *D. gilippus* and *D. eresimus* (Cramer), to sucking at dead or damaged parts of plants, or nectaring from plants that contain pyrrolizidine alkaloids (PAs). Owen [29] even recorded male *D. chrysippus* obtaining PAs at second hand by sucking at the bodies of moribund grasshoppers—*Zonocercus variegatus* L.—which had eaten PA-rich plants. 

All PAs are esters comprising a pyrrolizidine ring with one or two necic acid branch chains. They are acquired mostly from the families Boraginaceae (many genera), Apocynaceae (*Parsonsia* spp.), Fabaceae (*Crotalaria* spp.), and tribes Senecioneae and Eupatoriëae of the Asteraceae (many genera). Native PAs or their metabolites stored by insects are always initially plant-derived. All species examined from the nymphalid subfamilies Danainae and Ithomiinae contain PAs or their metabolites [30], males invariably with higher PA content than females [31,32]. PAs or derivatives are located in two body regions, in the androconial organs where they are key components—danaidone or danaidal—of the male courtship pheromone [33,34] and bound, either as native PA or their N-oxides, to chitin of the exoskeleton, especially in the wings [35]. As females visit PA-rich flowers less frequently than males, they obtain their PAs principally as nuptial gifts in the spermatophores they receive at copulation [31,32]. In turn, *D. gilippus* mothers pass PAs to their offspring via their eggs [36]. PAs are distasteful to potential invertebrate predators such as spiders which utterly reject them [37]; they and/or CGs may also provide protection from some species of ant. PAs are also potentially fatal liver poisons to vertebrates [38] but it is probably the combination of bitter taste and strong odor which most deters vertebrate predators [39], including birds, whose olfactory sense was once doubted [40], but has subsequently received the green light [41]. Thus, PA derivatives fulfil two vital functions, namely chemical defence and sexual communication: One of these is probably an exaptation which preceded or followed the other [42,43] but, given the ubiquity of both throughout the subfamily Danainae, precedence is an open question.

### 2.4. Courtship and Copulation

Those with experience of breeding *D. chrysippus* have long known that males denied access to PA plants were of no interest to females. The first thing males do after eclosion is to seek out PA plants from which they imbibe nectar or suck phloem sap from damaged parts, sometimes inducing leakage by leaf-scratching [44]. DASS has one record of a newly-eclosed male that spent his first 15 min on the wing supping PA-rich nectar from heliotrope flowers. Males less than two days old rarely succeed in persuading a potential partner to copulate unless she is sex-starved whereas, in contrast, old, battered males are eagerly sought after by females, possibly because their very longevity advertises long survival and hence genetic quality. 

Courtship behaviour in *D. chrysippus* was first described by Seibt and colleagues [45] and shown to be in all respects identical to that previously described by Brower and colleagues [46] in *D. gilippus*. The courtship pheromone manufactured in the paired alar glands in the hindwing comprises 19 known volatile ingredients, ten of which are unique to *D. chrysippus* [34] and no doubt comprise a species fingerprint. Long hours watching several *D. chrysippus* males unsuccessfully court a female *D. gilippus* left the firm impression that the hair-pencil pheromone is a specific identifier—though after 17 days one male succeeded [47]. 

Maturation of the dust-like particles known as pheromone transfer particles (PTC) requires prior contact between the alar glands and the androconial organs, known as hair-pencils, at the tip of the abdomen. The contact activity was first observed in idle male *D. gilippus* in Brazil by Fritz Müller [16] and many times since confirmed and experimentally verified. Under the sexual stimulus of chasing females, the hair-pencils become engorged with haemolymph and everted. The hair-pencils are coated with PTC which are transferred by a pursuing male onto the antennae of a female as he overtakes her. The female response is either to settle on the ground, indicating willingness to consider copulation—but not necessarily compliance—or to evade by flying away fast. Females deprived of male company—say in a breeding cage—are no ‘shrinking violets’ when introduced to males; they actively pester them for attention.

The sex life of *D. chrysippus* (and other danaines) is unusual among butterflies in two respects: The long duration of the copulation joining and its high frequency. *D. chrysippus* courtship begins around midday and the conjoined pair, carried in flight by the male, find a place to remain undisturbed in long grass or a tree. Copulation lasts an average of 3.5 ± 1.2 (range 0.7–5.0) hours [9]; in its final phase a spermatophore passes from male to female. The size of the spermatophore is very variable and it has been speculated—because so difficult to measure—that size is positively correlated with the duration of copulation. The content of the spermatophore includes sperm, both eupyrene (nucleated and functional) and apyrene (enucleate), together with defensive compounds such as CGs and PAs. It is known that the PA content of females is largely male-derived [31,32,48], while the enucleate apyrene sperm may function, among other things, as a nuptial gift of protein which allows her to produce more eggs [49]. Furthermore, in captivity both males and females have been observed to copulate on as many as four successive days and females may acquire up to ten spermatophores over a lifetime. Therefore, throughout her active sex life a female *D. chrysippus* [35,49] or *D. gilippus* [36] continues to garner male investment, thus boosting her chemical defences and, presumably, her life-span and egg production. Because males spend much time amassing PAs rather than pursuing females, the latter benefit from more unmolested time to search out suitable oviposition sites. 

### 2.5. Defence

From early days *D. chrysippus* has been regarded as a defended butterfly [15,50,51,52]. Early observers noted its unpleasant smell and the deterrent effect this had on potential predators [53], but the chemical underpinning of the olfactory defence by CGs [23,54] and PAs [9,31,35,48] was discovered much later (1965–present). As with many butterflies and other insects known or suspected to be chemically protected, *D. chrysippus* is boldly coloured in black, white and orange (Figure 1 and Figure 2), a feature termed aposematic by Poulton [55]. To be aposematic is to be memorable to would-be predators which learn, sometimes from a single unpleasant experience [56], to avoid further contact with such warningly coloured prey. Aposematic butterflies have long been known by entomologists as easier to catch because of their slow, gliding flight; these butterflies advertise themselves and appear to feel safe from molestation. Moreover, several observers have noted that aposematic butterflies carry evidence of beak damage on their wings more frequently than do supposed palatable species [57,58]. The argument, supported by observation, is that a bird (or lizard) that catches a distasteful butterfly grasps the wings and attempts to remove them before consuming the body, and in so doing registers the bitter taste, whereupon it releases the victim alive but with tell-tale wing damage. In contrast, an edible butterfly is consumed and leaves no evidence of its demise beyond perhaps wings discarded at the scene. No defence is perfect: One of us (IJG) recently saw a white-browed robin chat, *Cossypha heuglini* Hartlaub, attack a female *orientis* butterfly in Kigali and carry it away—this was the first attack he had seen on an African Queen in over forty years of field observations.

### 2.6. ‘Polymorphism’

Aposematic and distasteful insects are expected to be monomorphic, especially those that are mimicked, as is *D. chrysippus*, by many other species, both defended and undefended; the very good reason for the monomorphism is that if predators are to learn quickly that the butterflies are best avoided as potential meals the warning message needs to be bold, simple and invariable, as with any effective advertisement. Otherwise naïve predators could mistakenly attack, to the disadvantage of both parties. Throughout most of its range, the Queen, although divided into many geographical subspecies (Table 1), is monomorphic in each individual area as expected (Figure 2). 

**Figure 3:**
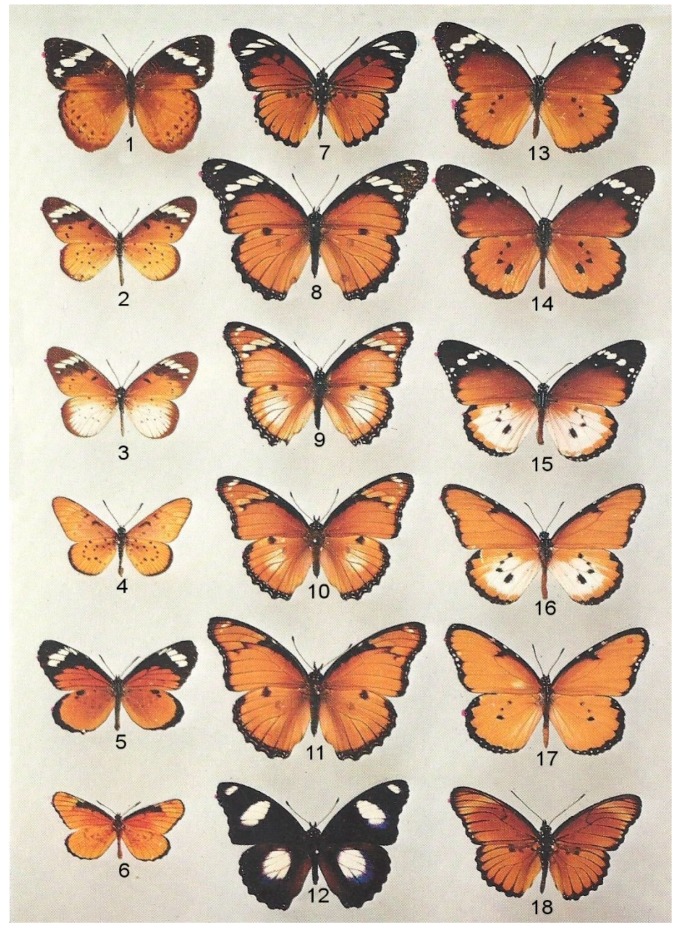
The comprehensive mimicry cycle based upon the colour forms of African *Danaus chrysippus*. 1. *Euriphene iris* Aurivillius (♀) (female-limited Batesian mimic of 13–14); Mpala, Congo Republic. 2. *Acraea encedon* L., form *encedon* (♀) (Müllerian mimic of 13–14); Kampala, Uganda. 3. *A. encedana* Pierre, f. *alcippina* Aurivillius (♀) (Müllerian mimic of 15); Falaba, Sierra Leone. 4. *A. encedon* f. *daira* Godman and Salvin (♂) (Müllerian mimic of 17); Nairobi Plains, Kenya. 5. *Mimacraea marshalli* f. *marshalli* Trimen (♂) (Batesian mimic of 13–14); Rusape, Zimbabwe. 6. *M. marshalli* f. *dohertyi* Rothschild & Jordan (♂) (Batesian mimic of 17); Nairobi, Kenya. 7. *Pseudacraea poggei* f. *poggei* Dewitz (♂) (Batesian mimic of 13–14); Solwezi, Zambia. 8. *Hypolimnas misippus* f. *misippus* (♀) (female-limited Batesian mimic of 13–14); Kibwezi, Kenya. 9. *H. misippus* f. *immima-alcippoides* (♀) (female-limited, imperfect Batesian mimic of 15); Ethiopia. 10. *H. misippus* f. *inaria-alcippoides* (♀) (female-limited Batesian mimic of 16); Sierra Leone. 11. *H. misippus* f. *inaria* (♀) (female-limited Batesian mimic of 17); Lake Mweru, Congo Republic. 12. *H. misippus* (♂) non-mimetic; Solwezi, Zambia. 13. *Danaus chrysippus* subspecies *orientis* Aurivillius (♀) (model); Durban, South Africa. 14. *D. chrysippus* ssp. *Orientis* (♂) (model); Muanza, Moçambique. 15. *D. chrysippus* ssp. *Alcippus* Cramer (♂) (model); Kitale, Kenya. 16. *D. chrysippus* hybrid f. albinus Lanz (♂) (model), Hoey’s Bridge, Kenya. 17. *D. chrysippus* ssp. *Dorippus* Klug (♀) (model), Nairobi, Kenya. 18. *Pseudacraea poggei* f. *carpenteri* Poulton (♂) (Batesian mimic of 17); Lulanguru, Tabora, Tanzania. Courtesy Oxford University Natural History Museum, Hope Department of Entomology [23].

**Figure 4:**
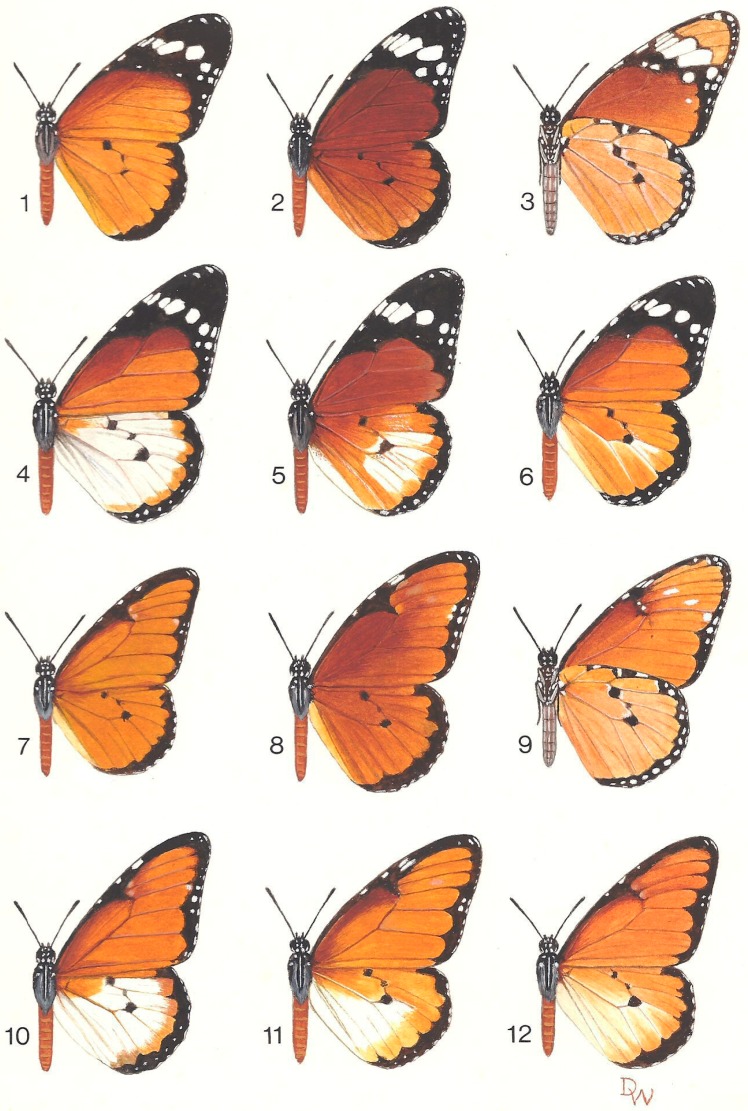
The African subspecies of *D. chrysippus*, including a range of hybrid forms. 1. *D. c. chrysippus*; 2. *D. c. orientis*; 3. *D. c. orientis* (underside); 4. *D. c. alcippus*; 5. Hybrid f. alcippoides, F1 from cross 2 × 4; 6. Hybrid f. alcippoides, F1 from cross 1 × 4; 7. *D. c. dorippus*; 8. Hybrid f. klugii, F1 from cross 2 × 7; 9. Hybrid f. transiens (underside), F1 from cross 1 × 7; 10. Hybrid f. albinus from backcross 4 × 11; 11–12. Hybrid f. semialbinus, F1 from cross 4 × 7. Courtesy Oxford University Natural History Museum, Hope Department of Entomology. Original painting by the late Derek Whiteley [14].

However, through eastern and central Africa, and only here, the species is not only ‘polymorphic’ (Figure 3) but intermediate hybrid forms are also numerous, omnipresent and fertile (Figure 4). In areas where the species has been extensively sampled and bred, such as Dar es Salaam, Kampala and Nairobi, it is known that the several African subspecies vary in frequency with season and the underlying reason for this is that all are migrants which come and go at different seasons [13]. Whether or not all the hybrid forms are similarly migratory is unknown but this, together with the comparative fertility of hybrids and their parents, are subjects of ongoing investigation. 

Although museum collections invariably include colour forms collected far distant from their likely origin, their frequency drops exactly as expected if isolated by distance [59]. Hence, the once paradoxical area of ‘polymorphism’ in East Africa is now understood to be essentially a contact zone where geographically distinct and largely allopatric subspecies (Figure 2), all of which remain mutually interfertile, meet seasonally in a manner that is replicated annually. This suggests that the four subspecies involved, *chrysippus*, *dorippus*, *orientis* and *alcippus* (Table 1), are in fact incipient species, especially as mate choice among them is strongly assortative in the field [60]. Quite remarkably, some of the many species that mimic *D. chrysippus*, both Batesian (*Pseudacraea poggii* Dewitz, *Mimacraea marshalli* Trimen) and Müllerian (*Acraea encedana* Pierre), are also polymorphic in East Africa (Figure 3) [23,61], whereas elsewhere, with the exception of the Batesian mimic *Hypolimnas misippus* (L.) [62,63,64,65,66]—unfortunately beyond the scope of this paper—they are not.

### 2.7. Genetic Control of Colour Patterns

The first evidence that f. chrysippus (Figure 4(1)) and f. dorippus (Figure 4(7)) are different forms of the same species was published by Poulton [67]. The first sustained attempt to investigate the genetics of the ‘polymorphism’ was made by Owen and Chanter [10] at Makerere University in Uganda. They showed that white hindwing, fixed in f. alcippus, (Figure 4(4)), is co-dominant with orange, fixed in ff. chrysippus and dorippus. Although heterozygotes for hindwing colour (Figure 4(5,6)) are mostly intermediate, hybrids are in a few cases indistinguishable from one or other parental type. Thus, dominance is variable. Smith [11] and Gordon [68] then showed, with butterflies from Dar es Salaam and Nairobi, respectively, that the f. chrysippus forewing pattern with the black, white-spotted apex (Figure 4(1–6)) is recessive to the plain orange pattern of f. dorippus (Figure 4(7, 10–12)). The heterozygote, f. transiens, (Figure 4(9)) is identifiable in 51.4 ± 0.02% (*n* = 1063) of cases (value updated here), the remainder being indistinguishable from f. dorippus. Finally, Clarke and colleagues [69], Smith [11] and Gordon [68] all identified a separate locus that governs the ground colour of the butterfly, either orange (Figure 4(1)) or brown (Figure 4(2)). Although easier in some places than others, with practice most intermediate *Bb* heterozygotes are identifiable by eye (Figure 4(5,8)). One possible caveat is that one of us (DASS) has recently found a population on Fuerteventura, Canary Islands, in which all individuals resemble *Bb* heterozygotes: This suggests there may be a third B allele intermediate in expression between *B* and *b*.

Smith [11] labelled the white hindwing locus A, with alleles *A* (non-white) and *a* (white); the ground colour locus was labelled B, with alleles *B* (brown) and *b* (orange) and the forewing pattern locus C, with alleles *C* (f. dorippus) and *c* (f. chrysippus). The A locus assorts independently of B and C. We have recent (unpublished) evidence for a second unlinked locus conferring brown ground colour but its geographical distribution and mode of interaction with the BC loci are as yet unknown. A nomenclature for the genotypes is given in Table 2.

The B and C loci were found to be linked with a recombination value of 2.7 ± 0.1 cM, *n* = 14 broods, and 258 offspring (value updated here). The recombination estimate is based only on males as meiosis in female Lepidoptera is achiasmate and crossing-over therefore absent [70,71,72]. Our recent work supports the hypothesis that B and C are two separate genes linked via complex rearrangements on chromosome 15 [73]. The combination of dominant alleles in coupling—*BC*—has not been found in any males from which we have bred, whereas one of the other three combinations is fixed in each of the subspecies, *chrysippus* (*bc*), *orientis* (*Bc*) and *dorippus* (*bC*). 

### 2.8. All-Female Broods

Owen and Chanter [10] first observed all-female broods reared from wild-caught females at Kampala, Uganda; 11/20 families raised were all-female. These authors concluded correctly that female excess they found in wild polymorphic populations was probably occasioned by the presence among them of two types of female, one producing only female offspring, the other broods that segregated 1:1 for sex. As all-female brooding was discovered long before bacterial endosymbionts, which cause (among many other detrimental effects) male killing in insects [74], Owen and Chanter assumed that all-female broods resulted from meiotic drive for the W chromosome [75]. When the sex ratio of the butterflies was further investigated at Dar es Salaam, Tanzania, by DASS [76] and near Nairobi, Kenya, by IJG [68], their observations, brought together in [14], established that: (1) Mixed broods average twice the size of all-female broods; (2) male larvae in all-female broods die at the point of hatching—known as early male killing (MK); (3) all-female brooding is heritable matrilinearly and invariably features male death, never meiotic drive; (4) in *D. chrysippus* there is a statistical interaction between the BC locus and brood type as the female offspring of hybrid all-female broods almost invariably carry the *bc* colour allele, its allelic partner being discarded in dead males; and (5) an all-female line occasionally reverts to a bisexual one [76], and vice versa, thus implying either the existence, at least in the Dar es Salaam population, of either a resistance mechanism [14] or failed transmission of an infective agent (see below). 

The cause of male-killing was finally established when Jiggins and colleagues [77], working in exactly the same part of Uganda as had Owen and Chanter, identified the endoparasite in *D. chrysippus* as a bacterium close to *Spiroplasma ixodetis* and detectable by PCR; the long delay in its discovery was because endoparasites cannot be cultured in vitro and thereby identified by obedience to Koch’s postulates. However, it has been shown [73,77] that *Spiroplasma*-infected caterpillars can be ‘cured’ by feeding them on leaves which have been painted with a weak solution of the antibiotic tetracycline. We have shown by PCR that cured females are *Spiroplasma*-free and that their ability to bear sons is thereby restored [73]. Jiggins’ discovery of *Spiroplasma* was confirmed by PCR in Kenya [78] and in Uganda [79]; no other causative organism has been found in subsequent searches. Direct PCR screening found a *Spiroplasma* infection rate of 40% for Kampala [77] compared to, by deduction from frequencies of all-female broods obtained from wild females, 30% for Dar es Salaam and up to 95% for some parts of Kenya. At one of our study sites, Kitengela near Nairobi, males have been absent from samples for as long as six months (see below), and yet, remarkably, nearly all females manage to acquire at least one spermatophore. We must speculate that rare visiting males escaped detection. It is notable that we have no evidence for MK outside the contact zone in East Africa. Furthermore, screening by PCR for *Spiroplasma* outside this area has proved overwhelmingly, though not absolutely, negative (Table 3). Our evidence then is that *Spiroplasma* and MK are tightly, perhaps even obligately, linked [73]. 

We confess that the vast areas where *D. chrysippus* is monomorphic are grossly under-sampled and we intend to correct that; nonetheless, the tight correspondence between MK and lengthy contact among differentiated subspecies suggests speciation and that it is this contact situation that has opened the gate for *Spiroplasma* to invade. Speciation is indirectly supported by assortative mating among subspecies *chrysippus*, *dorippus* and *orientis* wherever their males are sufficiently common [60] and at the B locus in ssp. *alcippus* in Ghana [68]; furthermore, there are Haldane effects in female hybrids [81] and the recent discovery of sex chromosome evolution [80,82,83], which often accompanies speciation [84,85].

### 2.9. The neoW Chromosome

**Figure 5:**
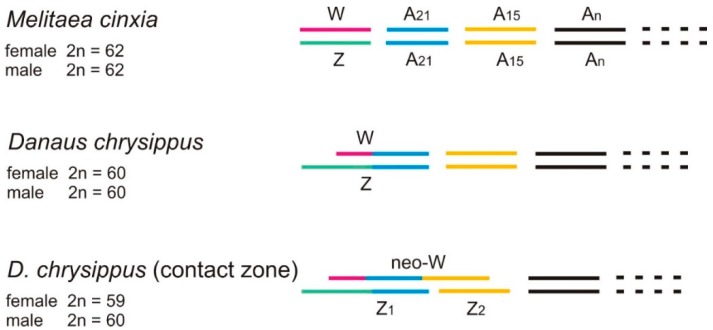
Composition of the sex chromosomes in the nymphalid model species *M. cinxia* and *D. chrysippus*. *M. cinxia* has the basic lepidopteran chromosome complement of W, Z and 30 pairs of autosomes (W, Z, A_2_, … A_31_). In *D. chrysippus* the Z chromosome is a product of a fusion between the ancestral Z and a homologue of *M. cinxia* A_21_. Its partner A_21_ has become the new W chromosome together with little or no part at all of the ancestral W chromosome. Variant females in the contact zone have neo-W chromosomes derived from a recent fusion between the *D chrysippus* W and a homologue of A_15_, and, hence, two Z chromosomes, Z_1_ and Z_2_. Z_1_ is the original *D. chrysippus* Z chromosome, Z_2_ the free homologue of A_15_.

We had noted occasional association between sex and genetic segregations at the BC locus since 1972 [14,63,68,86] and all recorded cases to date had occurred in butterflies from the contact zone in the environs of Nairobi or Dar es Salaam. However, W- and Z-linkage are impossible to distinguish by classical genetic analysis in single families and, as W-linkage is much the rarer, we long assumed Z-linkage. Eventually, an opportunity to cross African with Indian *D. chrysippus* in 2006 produced a single brood that was sex-linked for the BC locus and this prompted us to re-examine previous rare cases. In just one lineage we were able to prove by classical analysis that W-linkage of the BC locus was the correct interpretation [80] and this has since been amply confirmed both cytologically [83] and from DNA sequencing [73]. We found karyotypes (25 counts) for both sexes of *D. chrysippus* from South-east Asia, Israel, South Africa, Ghana and several sites in Kenya to be 2*n* = 60 [8]. These scores agreed with previous ones for male *D. c. chrysippus* in India [87] and male *D. c. alcippus* in Sénégal [88]. Males from Kitengela in the contact zone were also 2*n* = 60 [8]. However, in females from a MK line and known carriers of the *bc* chromosome from Kitengela (7 counts) we observed that in meiosis there were 28 bivalents and one trivalent (Figure 5), which we interpreted as comprising 56 autosomes, two Z chromosomes (Z_1_ and Z_2_) and a neoW chromosome (2*n* = 59). The latter was formed by fusion of the *bc* autosome with the ancestral W. The *bc* autosome is a homologue of the *Melitaea cinxia* L. (Glanville Fritillary butterfly) chromosome 15 [73]. This fusion explains the linkage of colour pattern and sex which is apparently confined to the contact zone in East Africa. Presumably, the W-autosome fusion was a rather recent event since in the representation analysis the genes in the W*bc* chromosome showed little signs of the decay which normally characterises butterfly W chromosomes [83]. This conclusion is reinforced by data [73] showing that the autosomes, with the notable exception of the *bc* autosome, of all four African subspecies show little evidence of divergence.

## 3. On-Going Hybrid Studies in the Nairobi Region, Kenya

### 3.1. Introduction

**Figure 6:**
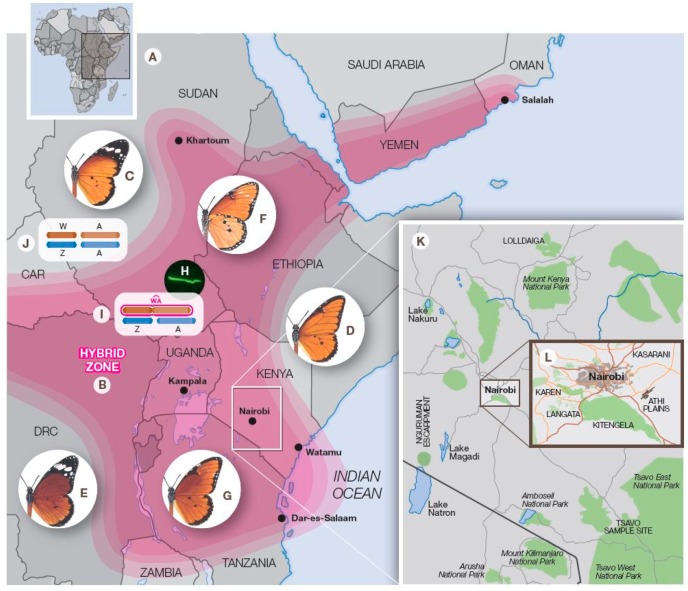
Map of Africa (**A**) showing the location of samples, approximate boundaries of the hybrid zone (**B**), and distribution outside the hybrid zone of subspecies *chrysippus* (**C**), *dorippus* (**D**) and *orientis* (**E**). Hybrid forms transiens (underside) (**F**) and klugii (**G**) are confined to the hybrid zone, as are the endosymbiont *Spiroplasma ixodetis* (**H**) and the fused neo-W karyotype (2*n* = 59) (**I**). Outside the hybrid zone the wild-type unfused karyotype (2*n* = 60) of all subspecies is shown in (**J**). Sampling sites mentioned in the text are shown in the insets, southern Kenya (**K**) and the environs of Nairobi (**L**). Acronyms: CAR = Central African Republic; DRC = Democratic Republic of Congo. Abbreviations: A, autosome (neoZ or Z_2_ in I), W, W chromosome; Z, Z chromosome (Z_1_ in I).

The East African contact zone of *D. chrysippus* (Figure 6) is characterised by the coincidence of two phenomena inside the area—but not outside it—which are individually unexpected, but in concert have no known parallel in the Animal Kingdom. The first bizarre feature is the ‘polymorphism’ for warning colour patterns, which makes little biological sense in a chemically protected species. Moreover, there is good evidence that mate choice among the subspecies of *D. chrysippus* is normally assortative for colour form [60,68], another indication that the so-called ‘polymorphism’ is not a within-species phenomenon but rather indicates incipient speciation. Furthermore, the frequency of each colour gene has a seasonally variable phenology that is, where studied at individual sites, replicated annually [13]. This indicates that allochronic migration—rather than natural selection or genetic drift—underlies annual rhythms of genetic change. The second phenomenon confined to East Africa is the evolution of the neoW chromosome which links the *bc* allele with the W chromosome, thus linking sex determination and colour pattern variation in *D. c. chrysippus*, a characteristic absent from the other three African subspecies. The neoW chromosome is nearly or absolutely fixed in *D. c. chrysippus* inside the contact zone but it appears that its range does not extend further, even within this subspecies. The W*bc* (neoW) female variant not only confers susceptibility to *Spiroplasma* infection but its carriers are almost invariably so infected, whereas outside the contact zone we have found only two infected females, one from South Africa and the other from Ghana. Moreover, where studied, all-female broods have never been found outside the contact area. We have suggested above that the association between MK and the neoW creates a ‘magic trait’ [80]. ‘Magic traits’ exist when pleiotropic gene(s) or a set of tightly linked genes control or influence both mate choice and a character under divergent selection [89]. In the case of the *D. chrysippus* contact zone sex, survival and colour pattern are inextricably linked through the neoW and its vertical co-inheritance with *Spiroplasma* in the egg. There is divergent selection based on larval resource competition and wing colour pattern, and there is non-random mating based on different genotype frequencies in males and females, and on assortative mating for colour pattern. Furthermore, there is almost zero possibility of recombination, making any distinction between a genuine ‘magic trait’ (involving pleiotropy) and a mimic ‘magic trait’ (involving linkage) [90] irrelevant. There are likely to be other traits influencing mate choice and survival, as yet undiscovered, that are similarly controlled by genes within the MK-neoW complex. We therefore formally identify this package as a matrilinearly-transmitted ‘magic trait’ which ensures that populations are female-biased throughout the east African contact zone [10,14], and which reaches its highest frequencies in the Nairobi region of Kenya [13]. Occasionally such neoW females produce progenies balanced 1:1 for sex in which all female offspring carry the W*bc* chromosome and all males have the alternative BC allele—*Bc* or *bC* on Z_2_. We have been unable to test a brood of this type for *Spiroplasma*, hence cannot yet distinguish between the alternative hypotheses that in such cases transmission of *Spiroplasma* has failed or a gene resistant to MK was introduced through the paternal line.

### 3.2. The Athi Plains

While teaching at the University of Nairobi in the years 1986–1994 one of us (IJG) made several collections of *D. chrysippus* eggs from the host plant *Gomphocarpus fruticosus* (L.) W.T.Aiton (Apocynaceae) in the Athi River Plains near Nairobi, a site since built over. Larvae from eggs which hatched successfully were fed on *Asclepias curassavica* L. and reared to adult in the laboratory. The collection of eggs rather than larvae or flying adults ensured that sex ratios would be unbiased. Whereas all collections were female-biased the sex ratios were very significantly variable around a mean of 74.5% female (Supplementary Table S1). Collections in January and February averaged 88.0% female against 62.6% for other months. It is also clear (Supplementary Figure S1A) that colour gene frequencies in the Athi River collections were subject to substantial seasonal change. For example, the *aa* genotype, though always uncommon, peaked in July/August and was least frequent in November. In contrast, the *bb* genotype was abundant from January through April and least frequent in July/August. Frequency of the *cc* genotype was bimodal being maximal in January and July/August. All these seasonal differences are statistically significant and far in excess of any changes that could be caused by natural selection or genetic drift. Instead they suggest substantial seasonal movements of populations. More comprehensive monthly data from Dar es Salaam, Tanzania, for 1972–1975 (Supplementary Figure S1B) show that the migration of subspecies *orientis* and *dorippus* follows a pattern that is broadly replicated on an annual basis [13]. These data implying widespread migration have been confirmed visually, or from short-term changes in mitochondrial DNA frequencies, on several occasions [29,91,92,93].

### 3.3. Kasarani

The Kasarani experiment [94] executed in two phases—April–July 2007 and March 2009–February 2010—was set up to investigate the apparent paradox that, whereas MK in butterflies is strongly associated with batch layers [73,95], *D. chrysippus* is not a batch layer; hence, the question arose concerning how populations of the butterfly inside the East African contact zone are advantaged by MK. Batch laying favours MK endosymbionts because infected female larvae can cannibalise dead brothers and do not have to compete with them for larval resources. Thus, both *Spiroplasma* and infected females increase their numbers at the expense of males and non-infected females. However, it is more difficult to envisage such a selective advantage for a bacterium that causes MK in a species such as *D. chrysippus* that lays its eggs singly. We investigated this paradox at Kasarani, stimulated by frequent observations by independent researchers (IJG, DASS, Rf-C) that *chrysippus* females lay preferentially on small isolated plants, often in numbers that exceed the ability of the plant to sustain them through to the pupal stage. 

As the spacious campus of the International Centre for Insect Physiology and Ecology (ICIPE) at Kasarani had no wild milkweed, to obtain eggs from *D. chrysippus* we used potted plants to attract oviposition by itinerant females from nearby populations. We generated an artificial cluster/isolate situation using *Asclepias curassavica* plants, a known favourite of *D. chrysippus*, on which transient females would lay eggs. The plants were arranged around the campus in tight clusters and scattered isolates (as detailed in reference [94]). Clusters and isolated plants were all well separated. Plants from both clusters and isolates were treated in two ways; half were left in place throughout the experiment, hence eggs and caterpillars were exposed to predators and parasitoids, while the other half were removed to the laboratory after three days. As expected, isolates attracted far more eggs (6.8 per plant) than clumped plants (2.0 per plant). In 2009–2010 all eclosed butterflies were tested by PCR for *Spiroplasma* infection.

The two phases of the field experiment encompassed contrasting levels of *Spiroplasma* infection. It was extremely high (95%) in the first phase (March–June 2009, *n* = 59), when not a single male was recorded, but fell during the second phase (July 2009–February 2010, *n* = 113) to an average of 54%, when average male frequency increased to 46%. The changes in infection rate had highly significant effects on egg survival to adulthood. When infection rates were high, 24% of eggs made it to adulthood, whereas when they were low only 8% survived. These changes reflect the gains realised through MK, which paves the way for females to predate dead male eggs and reduces competition for scarce larval foodplant on isolated plants. There was also a negative regression of adult body size on egg density on isolated plants, as expected under resource competition for larval foodplant.

### 3.4. Kitengela

The search for an African Queen population close to Nairobi that was not under immediate threat from development was rewarded in 2011 when IJG discovered Kitengela. Although described as a ‘farm’ by its owners Nani Croze and Eric Krystall, 90% of the 5 ha site is in reality lightly grazed savannah that is a perfect wild habitat for *D. chrysippus* throughout the year. Field and laboratory studies carried out during 2013–2015 established the following [8]:

(1) The population is ‘polymorphic’ for ssp. *Dorippus* (*CC*) and ssp. *Chrysippus* (*cc*). However, from the data in Table 4 it is clear, first, that the genotype arrays in two sexes differ sharply and indicate that they come predominantly from different source populations; secondly, much the most frequent female genotype is the hybrid form transiens (*Cc*).
(2) The occasional arrival of males in numbers following substantial rains, unaccompanied by females of the same genotype, suggests that the migratory behaviour of the two sexes differs sharply [96].
(3) Mating preference for C locus genotype was absent in both sexes, probably because the majority of males were *CC* and females *Cc*; thus, choice was severely curtailed.
(4) It follows from (1) and (3) that pairing is disassortative (negatively non-random) for genotype (Table S2, *p* < 0.00001) since shortage of males effectively eliminates female choice, while male choice is restricted because 83% of females are transiens (*Cc*). Thus, ‘choice’ is in practice denied to both sexes.
(5) Spermatophores per female averaged 1.7 (*n* = 260), while only 7.3% of females were unmated. These data mean that all females eventually find a mate. In a 1:1 population from Ghana, free from MK, the spermatophore/female average was 3.5 (*n* = 20) [8].
(6) Through a mark-recapture study in May–July 2015, 63.4% of all males caught were in copula compared to only 13.4% of females. The implication of the sex difference is that, if all females are eventually mated (see 5), the average male must mate 5 times, but to achieve 1.7 spermatophores per female, males must mate 8.5 times, hence, neoW females are likely to receive under-sized spermatophores from over-taxed males.
(7) The butterfly is a permanent resident varying in density between a minimum of 5.4/ha in October 2014 after a long drought and 68.8/ha in May 2015 following heavy rain.
(8) The mean sex ratio over three years was 84.1% female, varying between extremes of 100% female in the driest periods, up to six months duration, down to 72.3% female in May 2015. The mean sex ratio at Kitengela is the highest recorded for *D. chrysippus* anywhere and exceeds the long-term (30-year) average of 74.5% for the Nairobi region which includes places such as Athi Plains and Kasarani.
(9) The *Spiroplasma* infection rate of females at Kitengela, assessed by PCR, was 87.9% (*n* = 72), an estimate that tallies well with sex ratios and male-killing frequencies throughout the Nairobi region and over the years.

## 4. Discussion

The area of East Africa in which the ranges of the four subspecies of *D. chrysippus* overlap has a turbulent geological history which contrasts sharply with other regions of similar equatorial latitude such as West Africa, the Neotropics and South-east Asia. Whereas all three latter areas are low altitude and blanketed (until recent times) by evergreen rain forest, this habitat is closed to *D. chrysippus* and its Neotropical sister *D. gilippus*. Instead, in East Africa, the equatorial area has been slowly uplifted 1.5 km above sea level over some 30 m years [97] and is now a mosaic landscape vegetated by evergreen forest, lightly wooded savannah, dry scrub or semi-desert [98]. The climatic contrast between East Africa and other equatorial tropical regions is stark, the former being uniquely higher, cooler, drier and sunnier. It is this quarter of Africa that has over the past 5 m years seen an explosion of ungulate diversity [99] and all the early stages of hominin evolution [100]. It is also the meeting place of many polymorphic bird [101] and butterfly species which had evolved subspecific or morph-ratio differences either side of formerly forest east-west or north-south exclusion zones; examples abound, among them including the butterflies *Papilio dardanus* Brown, *Amauris niavius* (L.), *Hypolimnas anthedon* Doubleday, *Acraea encedon*, *A. encedana* and *Pseudacraea eurytus* (L.). 

The geological forces at work in East Africa have been twofold. Firstly, the 50 m-year Cenozoic cooling trend, which culminated 2.6 m years ago with the onset of the current series of ice ages, was caused by the collision of the Indian and Asian plates, and the subduction of the former to throw up the Himalayas. The powerful monsoon system created by the elevated Tibetan Plateau drew moisture away from East Africa [102]. Moreover, around 3–4 m years ago the northwards drift of Australia and New Guinea closed an ocean channel known as the Indonesian Seaway, which blocked the westward flow of warm Pacific waters into the Indian Ocean, thereby cooling it [97]. Both events contributed to East African aridification [103]. Secondly, starting around 30 m years ago, north-east Africa has been uplifted to such an extent that the stretched continental crust developed major north-south rifts and extensive volcanism. The eastern rift formed through Ethiopia, Kenya, eastern Tanzania and Mozambique, while the western branch cut through Uganda, the Congo, Rwanda/Burundi and western Tanzania [102]. The inter-rift area is almost completely congruent with the *D. chrysippus* contact zone and the distribution of the neoW chromosome in the female. It is thus tempting to postulate that the two events might be causally linked. A major consequence of the uplift and its accompanying mountainous ridges [103] was to cast a rain shadow over much of the inter-rift area and intensify its aridity [104], especially over the last 3–4 m years [97]. From 17.3–14.7 k years ago, Lake Victoria, currently the world’s largest freshwater lake, completely dried up and rain forest was restricted to small ‘island’ refugia in the western half of Africa [105,106]; dry savannah and extensive desert dominated most of the continent. In particular, a corridor stretching from Somalia to Namibia was arid throughout the Pleistocene. Thus, it is likely that through most of the Late Pleistocene *D. chrysippus* was panmictic throughout the continent. 

For a short time in the early Holocene, from around 13–7 k years ago, high lake stands throughout Africa indicate that conditions became suddenly humid [107,108], even the Sahara and Kalahari Desert regions becoming grassland with scattered trees [105]. As Moreau memorably commented, the present Congo rain forest grows on Kalahari sand. Under these conditions rain and montane forest rapidly increased their ranges to merge and produce a continuous west–east equatorial belt across the continent [109,110,111], with a northerly extension stretching possibly as far as the Tibesti Mountains. The previously panmictic *D. chrysippus* population was thereby divided into a number of groups isolated by forest. It is probable that for some 6 k years in the Holocene pluvial period *D. chrysippus* was split into separate populations north and south of an equatorial forest belt and diverged in isolation. There is evidence that the brief pluvial period ended rather suddenly to be replaced around 7 k years ago by much drier conditions [112,113,114,115]. The fast contraction of forest cover would again open up the continent allowing once isolated savannah species to mix and hybridise. From this, we propose that geographical isolation of *D. chrysippus* populations, caused principally by afforestation in the pluvial period, ended around 7 k years ago as savannah rapidly displaced forest. This would allow once isolated populations that had diverged in isolation to re-establish contact on a permanent or semi-permanent basis and thereby initiate a hybrid zone. It is possible that rapid changes in distribution of the *D. chrysippus* colour forms over short time-frames accounts to some extent for the notorious failure of the polymorphic Batesian mimic *H. misippus* (Figure 3) to keep up with its model [63,64]. 

The distinct and largely discrete geographical ranges of the four African colour forms of *D. chrysippus* at the present time (Figure 2) in themselves argue that speciation has been under way. A supposition that all four forms are migratory is supported both by direct observation [91,92] and the phenology of colour gene frequencies in places such as Dar es Salaam and Nairobi where populations have been monitored over the long term [13,93]. The widespread occurrence of all four forms outside their heartlands (Figure 2) testifies to their dispersal ability. Abrupt invasion into a female-biased population by males of different genotype, as at Kitengela, seemingly occurs in all populations subject to MK within the contact zone. We deduce that males are more dispersive than females, not only from their numerical superiority in most field samples of adults, but also by mark-recapture data and their invariably having longer wings [81]. We plan to test this contention further by wing measurements with much larger samples and by comparing stable isotope compositions of the two sexes: The more migratory male is expected to be the more variable sex as it will have inhabited a wider variety of environments. The negative non-random mating that follows male invasion at Kitengela (Table S2) is paradoxical since, given adequate numbers of each colour form available to mate in each sex, mate choice is invariably positively non-random, i.e., assortative [60,68]. It appears that if, as at Kitengela, the two sexes predominantly differ in genotype and choice is effectively denied, disassortative mating is the inevitable outcome [86]. Throughout the hybrid zone, wherever MK occurs, colour gene frequencies differ between the sexes [13,14,116], this being the case, wherever such genotype differences become extreme, positive non-random mating will be reversed [86] and heterozygotes, mostly female, will far exceed Hardy–Weinberg expectations [13].

Although all four African forms of *D. chrysippus* are mutually inter-fertile, as are their F1 offspring, F1 heterozygotes, especially females, are generally smaller [81]; as the female is the heterozygotic (ZW) sex, it is as expected the more affected and denotes Haldane effects [81,117]. In MK broods, the surviving females suffer several disadvantages. In each generation they lose half their genes in dead sons, invariably including both paternal Z chromosomes; all their daughters inherit the ‘magic trait’ package [mitochondria + *Spiroplasma* + neoW chromosome] which consigns them and their daughters in perpetuity to a genetic ‘black hole’. Because males are always scarce the effective population size (*N_e_*) is severely reduced and could lead to inbreeding. At Kitengela, where the average female shares a male partner with four other females, their daughters are half-sisters. Further disadvantages are that MK females are smaller [78] and have limited access to males, hence, to paternal investment of defensive chemicals and protein in their spermatophores. A compensating trade-off is that high (95%) *Spiroplasma* infection (at Kasarani) improves survival of ‘magic trait’ females from egg to adult, presumably because removal of males alleviates competition for forage. As the distributions of *Spiroplasma*, MK and the neoW chromosome are limited to the Rift Valley contact zone, this means that ‘magic trait’ females are relatively static and wholly dependent on incoming migratory males for mates. Since the sexes in female-biased populations are largely unmatched for genotype, the persistence of dysfunctional colour polymorphisms is assured.

## 5. Conclusions

It seems that a genetic ‘black hole’ between sspp. *chrysippus* and *dorippus* was initiated around 2.2 k years ago [73] in the region around Nairobi when ssp. *chrysippus* was invaded by *Spiroplasma*. At around the same time chromosome 15 of *chrysippus* became fused with the W chromosome in the *chrysippus* female [73,80,83]. The neoW chromosome paired at meiosis with both the ancestral Z (which became Z_1_) and the male’s chromosome 15 (which became Z_2_) [80,83]. As *Spiroplasma* caused MK, all males which mated with neoW females suffered the disadvantage of losing both Z chromosomes and half their autosomal genome in her dead sons. In the early stages the incoming males would mostly be *dorippus* (*bC*/*bC*) but, as the neoW mutation expanded through the contact zone, a similar fate eventually befell males of all other subspecies. The implication of these mutations is that over half the male genome, including the entire paternal portion of it (which includes the BC colour locus), is lost in each generation. We contend, however, that reduced resource competition must have exceeded any costs to infected females to have allowed the neoW lineage to have spread as it has. The fusion and invasion events must have been nearly simultaneous since we have found nearly perfect matrilineal linkage of the neoW, mitochondria, *Spiroplasma* infection and MK [73]. 

Occasional male survivors in otherwise female-dominated broods, and even rare 1:1 broods that are sex linked for colour genes [13,76] probably result from receiving an endoparasite titre insufficient to kill the males. The main result of the several linked changes was the creation of populations dominated by hybrid neoW females which are almost entirely dependent for mates on wandering migratory males of different genotype. The genetic effect is the erection over the last 2.2 k years of a reproductive barrier to gene exchange between neoW females and males of all four subspecies. However, following the retreat of forest some 7 k years ago, panmixia would be re-established for some 5 k years before the neoW-MK mutation appeared and this is reflected by the virtual absence of population structure outside the colour-patterning loci [73]. The impact of the spread of the neoW females and the consequent reproductive barrier has not yet left a clear signal in the form of population structure.

Looking south to Dar es Salaam (Figure 6) where the frequency of *Spiroplasma* is only 30% or so, the introgression barrier affects crosses between neoW females and *chrysippus*, *dorippus* or *orientis* males [118]. Crosses between *orientis* and *dorippus* are not affected because neither subspecies carries the ‘magic trait’. Towards the west at Kampala (Figure 6) *Spiroplasma* frequencies are around 40% and crosses between ‘magic trait’ females and any of *chrysippus*, *orientis* or *alcippus* males involve MK [10,76]. Northwards to Khartoum (Figure 6) where the neoW-MK trait is absent, *chrysippus* × *alcippus* crosses are not affected by MK [119]. Although more data across the contact area and immediately outside its boundaries are much needed, present evidence supports the notion that reproductive barriers exist between neoW females and males from all four subspecies. High frequencies of the ‘magic trait’ in *bc*/*bc* females ensures that *bc*/*bc* males are scarce inside the contact zone. Whereas in crosses among subspecies which lack the ‘magic trait’, e.g., *dorippus* × *orientis*, *dorippus* × *alcippus* and *orientis* × *alcippus*, there is no MK, Haldane effects are detectable in at least some cases [81] because female hybrids are relatively unfit.

Although correlation is not causation, the near-congruence of the areas circumscribed by the East African rifts and the hybrid zone, including the ranges of *Spiroplasma* infection, the neoW mutation and MK in *D. chrysippus*, is alluring. The estimated onset of the late Holocene dry period (7 k years ago) leaves a gap of around 5 k years until the estimated origin of the neoW-MK complex some 2.2 k years ago. Judging from present frequencies of *Spiroplasma* infection, the neoW-MK complex was first selected in the Nairobi region, where sspp. *chrysippus* and *dorippus* are in regular and prolonged contact, and subsequently spread outwards across the Rift, eventually to engulf crosses between neoW-MK females and males of all four subspecies. As *Spiroplasma* is absent or rare throughout the extensive range of *D. chrysippus* outside the East African hybrid zone, but widespread within it, the symbiosis between butterfly and microorganism inevitably restricts gene flow and is expected to expedite speciation. While seemingly inevitable in the long run, the absence of a signal that genomes have diverged—apart from the colour pattern regions under selection—infers that insufficient time has elapsed for speciation to become irreversible. It is even possible that once differentiated populations are in the process of re-integrating into a panmictic population. Nonetheless, speciation signals exist in the form of geographical divergence of colour patterns, assortative mate choice and Haldane effects. 

Whilst there are striking similarities between our system and that of *Hypolimnas bolina* (L.), where MK is driven by *Wolbachia*, there are also important and interesting differences. *H. bolina* has been largely studied on islands where the lack of immigrant males leads to all-female populations that are then threatened with extinction. Hence, there is strong pressure for suppressor genes. Indeed, on two Samoan islands the population sex ratio recovered from 100:1 (females:males) to near 1:1 in less than ten generations, although they are still infected by the same strain of *Wolbachia* [120]. Genomic data revealed a selective sweep that created a conserved region of ~25 cM surrounding the suppressor locus [121]. In contrast, the MK females in the contact zone of *D. chrysippus* do not form a self-contained population. Males of the Queen are drawn into the contact zone from east, west, north or south by seasonal movements of the Inter-Tropical Convergence Zone. Unlike an island, this therefore allows the largely female contact zone to persist via a regular supply of different immigrant males. So, there is no specific selection pressure for suppressors in this population. The MK hybrid matriline has thriven and expanded already for 2.2 k years, equivalent to 26 k generations. It forms a genetic sink, at least for the colour morph genes that mark the subspecies, and thus stabilizes the differences. The causative role of a microorganism with a putative speciation process, and especially its restriction to a hybrid contact zone, has no known parallel in the Animal Kingdom.

## Figures and Tables

**Table 1:** The taxonomic history of *Danaus chrysippus* in Africa.

Subspecies & Forms	Original Status & Authority	Present Status & Range
*chrysippus*	*Papilio chrysippus*, L. 1758	ssp, S. Europe, N. Africa, Asia
*alcippus*	*Papilio alcippus*, Cramer 1777	ssp, W. Africa
*dorippus*	*Euploea dorippus*, Klug 1845	ssp, NE Africa, Arabia
*orientis*	f. of *Danaida chrysippus*, Aurivillius 1909	ssp, S. Africa, Indian Ocean
klugii	*Limnas klugii*, Butler 1886	*orientis* × *dorippus* F1 hybrid
infumata	f. of *Danaida chrysippus*, Aurivillius 1898	*chrysippus* × *orientis* F1 hybrid
transiens	f. of *Danaida dorippus*, Suffert 1900	*chrysippus* × *dorippus* F1 hybrid
alcippoides ^1^	f. of *Danaus chrysippus*, Moore 1883	*chrysippus* or *orientis* × *alcippus* F1 hybrid
albinus	f. of *Danaida dorippus*, Lanz 1896	*alcippus* × *dorippus* F2 hybrid
semialbinus	f. of *Danaida dorippus*, Strand 1910	*alcippus* × *dorippus* F1 hybrid

f., form; ssp., subspecies. ^1^ This name is correctly applied to the Asian subspecies *alcippoides* [7] but also, traditionally, to *chrysippus* × *alcippus* and *orientis* × *alcippus* F1 hybrid forms in Africa. Note: The past failure to distinguish polymorphism from sub-speciation has spilled into nomenclature and caused much confusion. In Table 1, subspecies are italicised, e.g., *D. c. chrysippus*, *D. c. alcippus* etc., whereas visually identical phenotypes that occur outside their usual range and within that of an alien subspecies are unitalicized, e.g., f. chrysippus, f. alcippus etc.; unitalicized formats are also used for colour forms that segregate genetically within broods and for hybrid-only forms, e.g., f. klugii, f. albinus.

**Table 2:** Nomenclature and genotypes of the colour forms of *D. chrysippus*.

Genotype	*AA*	*Aa*	*aa*
*bc*/*bc*	chrysippus	orange alcippoides orange alcippus	orange alcippus
*Bc*/*Bc*	orientis	brown alcippoides brown alcippus	brown alcippus
*bC*/*bC*	dorippus	semialbinus albinus	albinus
*bC*/*bc*	transiens	semialbinus-transiens albinus-transiens	albinus-transiens
*Bc*/*bC*	klugii	semialbinus-klugii albinus-klugii	albinus-klugii
*Bc*/*bc*	infumata	alcippoides-infumata alcippus-infumata	alcippus-infumata

**Table 3:** Geographical comparison of sites giving evidence of *Spiroplasma* presence ^1^ in *D. chrysippus*.

Study Areas	*Spiroplasma*		
	+	−	*n*
East Africa	30 ^1^	0	30
Elsewhere	2 ^2^	12 ^3^	14
Totals	32	12	44

χ^2^_1_ = 34.764; *p* < 0.00001. ^1^ Evidence for *Spiroplasma* presence comprises either male killing (MK), positive PCRs for *Spiroplasma* or both; all MK sites are inside the contact zone in Uganda, Kenya or Tanzania. ^2^ *Spiroplasma* has been recorded at one site in each of Ghana and South Africa but there is no evidence of MK in either place. ^3^ Negative MK sites: Cape Verde Is., Ghana, India, Madagascar, Mauritius, Oman, Sierra Leone, South Africa, Zambia, and Zimbabwe [80].

**Table 4:** Observed frequencies of C genotypes (corrected for penetrance *) in 19 field samples of flying *D. chrysippus* at Kitengela, Kenya, May 2013–September 2015 [8].

Sex	Genotype Frequencies			
	*CC*	*Cc*	*cc*	*n*
Females	0	801	118	919
Males	130	37	7	174
Totals	130	838	125	1093

* The correction for penetrance for *Cc* in *C*—offspring is derived from laboratory breeding data in which 0.514 ± 0.015 (*n* = 1063) of known *Cc* heterozygotes are visually identifiable.

## Supplementary Materials

The following are available online at https://www.mdpi.com/2075-4450/10/9/291/s1, Figure S1: (**a**) Histograms showing the frequencies (per cent) of (A) females and (B–D) the three homozygous recessive phenotypes *aa* (B), *bb* (C) and *cc* (D) at Nairobi. Symbols on the co-ordinate: J = January, F = February, A = April, M = May, J/A = July/August, N = November. $\bar{x}$ = the mean value of the six samples [13]; (**b**) Frequencies (per cent) as three-month moving averages for the *cc* genotype (o) and females (•) in monthly samples of *D. chrysippus* from February 1972 to September 1975 on the campus of the University of Dar es Salaam, Tanzania. The approximate durations of wet seasons (dashed lines indicating periods that are variable) and the two monsoons (SE = south–east, NE = north–east) are shown at the top [13]; Table S1: Sex ratios of Danaus chrysippus collected as eggs, Athi River Plains, Nairobi, 1986–1994. (Expected numbers if the true sex ratio is stable at 74.5% female in parentheses); Table S2: Disassortative (negative non-random) mating for C locus genotype (expected numbers in parentheses if mate choice is random) in D. chrysippus at Kitengela, Kenya, May–July 2015.
