# Supplementary material for: Neo Sex Chromosomes, Colour Polymorphism and Male-Killing in the African Queen Butterfly, Danaus chrysippus (L.)
Source: Insects. 2019 Sep 9;10(9):291. doi: 10.3390/insects10090291 (PMC6780594; doi:10.3390/insects10090291)
Supplement: Supplementary file 1 [file insects-10-00291-s001.pdf]

**Table S1.** Sex ratios of *Danaus chrysippus* collected as eggs, Athi River Plains, Nairobi, 1986–1994. (Expected numbers if the true sex ratio is stable at 74.5% female in parentheses).

| Date | Males | Females | Totals | % Female | Deviation from Mean | $\chi^2_1$ |
|---|---|---|---|---|---|---|
| January 1986 | 5 (14.25) | 52 (42.75) | 57 | 91.2 | + | 8.005 |
| April–May 1987 | 66 (47.0) | 122 (141.0) | 188 | 64.9 | − | 10.241 |
| July–August 1987 | 20 (13.0) | 32 (39.0) | 52 | 61.5 | − | 5.025 |
| November 1988 | 24 (15.5) | 38 (46.5) | 62 | 61.3 | − | 6.215 |
| February 1989 | 20 (34.75) | 119 (104.25) | 139 | 85.6 | + | 8.348 |
| January 1994 | 8 (15.5) | 54 (46.5) | 62 | 87.1 | + | 4.839 |
| Totals | 143 | 417 | 560 | 74.5 | 0 | 42.673 |

Against a mean expectation that the sex ration is stable at 74.5% female, $\sum \chi^2_5 = 42.673$, $p$ for heterogeneity is <0.00001; thus, the sex ration is unstable and varies with season.

**Table S2.** Disassortative (negative non-random) mating for C locus genotype (expected numbers in parentheses if mate choice is random) in *D. chrysippus* at Kitengela, Kenya, May–July 2015.

| Genotypes | *CC* | *Cc* | *cc* | *n* |
|---|---|---|---|---|
| Males *in copula* | 32 (16.0) | 16 (27.5) | 4 (8.5) | 52 |
| Males free flying | 24 | 5 | 1 | 30 |
| Total males | 51 | 26 | 5 | 82 |
| Females *in copula* | 0 (16.0) | 39 (27.5) | 13 (8.5) | 52 |
| Females free flying | 0 | 282 | 53 | 335 |
| Total females | 0 | 321 | 66 | 387 |
| Total *in copula* | 32 (32) | 55 (55) | 17 (17) | 104 |
| Total unmated | 24 | 287 | 54 | 365 |
| Totals | 51 | 347 | 71 | 469 |

$\sum \chi^2_4$ for negative non-random mating = 46.328; $p < 0.00001$. All mate females carried the *Wbc* (neoW chromosome) and are *Cc* or *cc*, wherease a majority of mating males (61.5%) are *CC*. The sex ratio estimate (82.5% female) does not differ from the mean sex ratio for May–September 2015 of 81.2% female ($n = 531$). Given the mean spermatophore count of 1.7 for females dissected ($n = 260$), each male must mate on average 8.5 times.

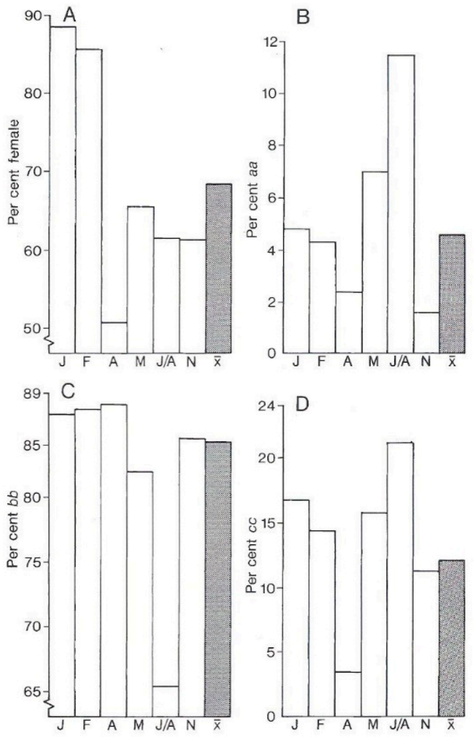


(**a**)

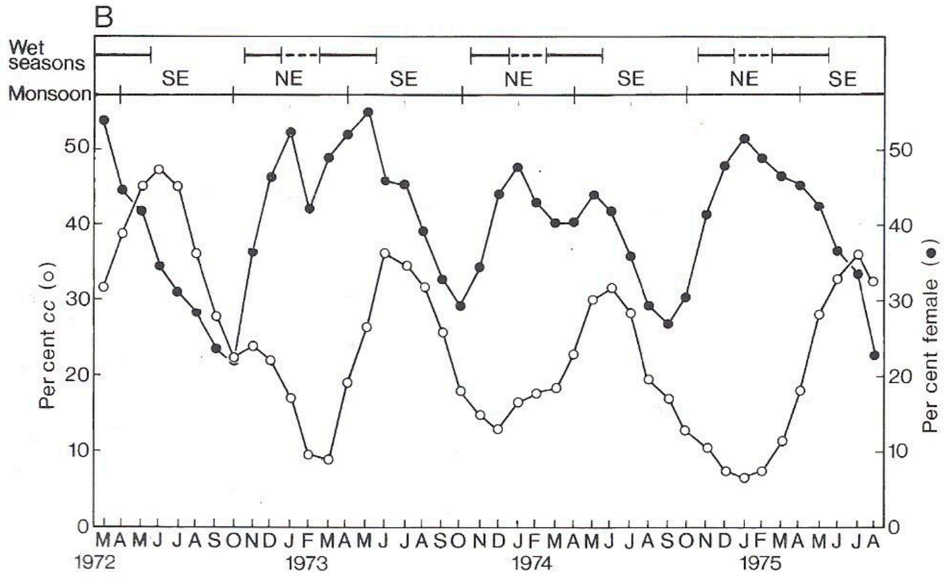


(**b**)

**Figure S1.** **(a)** Histograms showing the frequencies (per cent) of (A) females and (B–D) the three homozygous recessive phenotypes *aa* (B), *bb* (C) and *cc* (D) at Nairobi. Symbols on the co-ordinate: J = January, F = February, A = April, M = May, J/A = July/August, N = November. $\bar{x}$ = the mean value of the six samples [13]; (**b**)Frequencies (per cent) as three-month moving averages for the *cc* genotype (o) and females (•) in monthly samples of *D. chrysippus* from February 1972 to September 1975 on the campus of the University of Dar es Salaam, Tanzania. The approximate durations of wet seasons (dashed lines indicating periods that are variable) and the two monsoons (SE = south–east, NE = north–east) are shown at the top [13].
